# Inhibitory Effects of Culinary Herbs and Spices on the Growth of HCA-7 Colorectal Cancer Cells and Their COX-2 Expression

**DOI:** 10.3390/nu9101051

**Published:** 2017-09-21

**Authors:** Andrius Jaksevicius, Mark Carew, Calli Mistry, Helmout Modjtahedi, Elizabeth I. Opara

**Affiliations:** School of Life Sciences, Pharmacy and Chemistry, Kingston University, Penrhyn Road, Kingston upon Thames KT1 2EE, UK; k0622438@Kingston.ac.uk (A.J.); M.Carew@Kingston.ac.uk (M.C.); C.Mistry@Kingston.ac.uk (C.M.); H.Modjtahedi@Kingston.ac.uk (H.M.)

**Keywords:** colorectal, cancer, herbs, spices, COX-2

## Abstract

It is unclear if the anti-inflammatory properties of culinary herbs and spices (CHS) are linked to their ability to inhibit Colorectal cancer cell (CRC) growth. Furthermore, their therapeutic potential with regards to CRC is unknown. The aim of this study was to establish if the inhibition of HCA-7 CRC cell growth by a selection of culinary herbs and spices (CHS) is linked to the inhibition of the cells’ cyclooxygenase-2 (COX-2 )expression, and to investigate their therapeutic potential. CHS inhibited the growth of Human colon adenocarcinoma-7 (HCA-7) cells; the order of potency was turmeric, bay leaf, ginger, sage, and rosemary; their combinations had a synergistic or additive effect on cell growth inhibition. CHS also inhibited COX-2 expression and activity; this action was comparable to that of the specific COX-2 inhibitor Celecoxib. Coincident with COX-2 inhibition was the accumulation of cells in the sub G1 phase of the HCA-7’s cell cycle and, using bay leaf and turmeric, the cleavage of caspase 3 and poly (ADP-ribose) polymerase (PARP). This latter effect showed that the effect of these CHS on growth arrest was irreversible, and was comparable to that of the caspase activator Etoposide. This study provides evidence of a link between the inhibition of HCA-7 growth, and its COX-2 expression, by CHS, and their therapeutic potential.

## 1. Introduction

Colorectal cancer (CRC) is one of the most commonly diagnosed cancers in developed countries, and cases are rising in developing countries [1,2]. Inflammation, specifically chronic inflammation, plays an important role in the development of CRC [3,4]. One key mediator of the inflammatory response is the enzyme cyclooxygenase-2 (COX-2) and its product prostaglandin E2 (PGE-2), and both are known to promote carcinogenesis [5]. Moreover, it has been found that patient histological samples of CRC tumours have overexpressed COX-2 [6,7,8,9]. Furthermore, when this enzyme is targeted using non-steroidal anti-inflammatory drugs (NSAIDs), the risk of CRC has been shown to be reduced [10,11,12]. However, these drugs have adverse side effects, and hence safer alternatives are required [13,14]. 

There are numerous foods and food constituents that have been shown to possess anti-inflammatory effects [14,15], and culinary herbs and spices (CHS) are among them [16,17,18]. Although they are consumed in small amounts, these foods contain high levels of phytochemicals, especially polyphenols, which have limited bioavailability, suggesting that a significant part of their action may be limited to the gut [19,20]. Studies have shown that some CHS inhibit the growth of CRC cells, suggesting that these foods are of potential use in the prevention and treatment of CRC [21,22,23]. However, despite studies that show that polyphenolic constituents of CHS, primarily curcumin, are well established inhibitors of a key inflammatory mediator COX-2 in HCA-7 and HT29 CRC cells [3,24,25,26,27] there is little information on the effects of CHS. This paucity of information is despite the fact that there is a growing amount of interest in the bioactivity of whole foods, not just on their own but in combination, so as to know and understand more fully their beneficial potential [20]. Thus, the aim of this study was to investigate the effect of a selection of CHS (individually and in combination) on HCA-7 CRC cell growth, and its expression of COX-2, ascertain if these activities are linked, and determine if the CHS are of therapeutic potential with regards CRC.

## 2. Materials and Methods

### 2.1. Preparation of Culinary Herb and Spice (CHS) Extracts 

The CHS were purchased online from Neal’s Yard remedies (London, UK): bay leaf (*Laurus nobilis*), rosemary (*Rosmarinus officinalis*), sage (*Salvia officinalis*), ginger (*Zingiber officinale*), and turmeric (*Curcuma longa*). The selection of the CHS was based on preliminary potency studies. These CHS were extracted using a method adapted from Huang et al. [28] with some modifications. Briefly, herbs/spices, with the exception of ginger and turmeric, which were purchased in powder form, were ground up using a pestle and mortar and then 2 g of ground herb were added to a glass bottle and extracted in 25 mL of solvent (deionised water or 42% ethanol (*v/v*)). The bottles were then wrapped in aluminium foil and placed on an orbital shaker (Jeiotech OS-7100, Fisher Scientific, Loughborough, Leicestershire, UK) for 2.5 h Thereafter, the contents were transferred into a sonicator (Elmasonic S 10H ultrasonic bath, Fisher Scientific, Leicestershire, UK) and sonicated for 70 min at a frequency of 35 kHz. After sonication, the extracts were filtered using a two-stage filtration process: for Stage 1, the extracts were filtered using Whatman No. 1 filters (Whatman, Fisher Scientific, Loughborough, Leicestershire, UK) to separate the extract from any solid material and then to make the extract more pure for Stage 2 a Whatman No. 6 filter paper (Whatman, Fisher Scientific, Loughborough, Leicestershire, UK) was used. The filtered extracts were then aliquoted and stored at −80 °C. The extracts prepared were turmeric in ethanol (TE), ginger in ethanol (GE), bay leaf in ethanol (BLE), sage in ethanol (SE), sage in water (SA), rosemary in ethanol (RE), and rosemary in water (RA). The extracts prepared were based on preliminary potency studies as with the selection of CHS. The effect of combinations of CHS was also investigated to determine if they had any additive/synergistic effect. All the combinations used in this study were prepared by using half of the concentration of each extract that was used individually for the same experiment. The concentration of the extract was based on their phenolic content (μg gallic acid equivalents (GAE)/mL). Based on preliminary potency studies, the following combinations were used: rosemary in water and rosemary ethanol (RAE), sage in water and sage ethanol (SAE), bay leaf and turmeric ethanol (BLTE), sage and ginger ethanol (SGE), bay leaf and sage ethanol (BLSE), and rosemary and turmeric ethanol (RTE).

The total phenolic content for each herb and spice extract was determined using the Folin–Ciocalteu (F-C) colorimetric method used by Singleton et al. [29] and modified by Tang et al. [30]. To determine if any of the observations were related to the CHS’ polyphenol content or simply their weight, the concentrations of the extracts were expressed as as gallic acid equivalents (GAE) for the total polyphenol content, and dry weight (DW) equivalents (for 1 g of dry weight). 

### 2.2. Growth Inhibition Studies: Effect of Herb and Spice Extracts (Individual and in Combination) on Growth of HCA-7 Cells

HCA-7 CRC cells, obtained from Culture Collections Public Health England, were grown in Dulbecco Modified Eagle Medium (DMEM) (Sigma-Aldrich, Poole, Dorset, UK, D5796) supplemented with 10% foetal bovine serum (FBS) (Sigma-Aldrich, Poole, Dorset, UK, F7524) and antibiotics: penicillin (50 units per mL), streptomycin (0.05 mg/mL), and neomycin (0.1 mg/mL), at 37 °C, 5% CO_2_ atmosphere. The growth inhibition studies were first performed using the sulforhodamine B (SRB) assay, and the protocol was adapted from Khelwatty et al. [31]. In brief, confluent HCA-7 cells were trypsinised and re-suspended in DMEM with 10% FBS and then seeded on to 96-well plates (10,000 cells suspended in 100 μL of DMEM per well) and placed into an incubator for four hours. The CHS were then prepared using a doubling dilution technique; the starting concentration for each extract was 20 μg GAE/mL. Following the 4 h incubation period, the CHS extracts (100 μL) at various concentrations (individually and in combination) were added to the wells. Cells were treated with the extracts for 5 days until the control wells (DMEM containing 10% FBS only) reached confluence. Thereafter, the plate was fixed for 1 h with 10% trichloroacetic acid (TCA), then washed with tap water, dried and stained with sulforhodamine B (SRB) (0.4% (*w/v*) in acetic acid (1%, *v*/*v*) (100 μL per well) for 1 h Thereafter, SRB was removed and the stain was re-solubilised by adding 100 μL Tris-base (10 mM) into each well, and absorbance was then read at 565 nm using an Epoch microplate reader (Biotek, Swindon, UK). 

To determine if the effect of the CHS was cancer cell specific, the effect of some of the most potent CHS on the growth of normal cells, human foreskin fibroblasts (HFF-2 cells) donated by Dr. Chioni (Kingston University London, Kingston upon Thames, UK) and were a gift from Dr. Richard Grose (Queen Mary, University of London, London, UK) was also investigated, using the same protocol described above for the HCA-7 cell line. 

### 2.3. The Effect of Culinary Herb and Spice Extracts on COX-2 Expression in HCA-7 CRC Cells 

Based on the growth inhibition studies, described above, specifically the potency of the extracts RE, SE, BLE, GE, TE, RTE, BLSE, SGE, and BLTE were chosen to study their effect on COX-2 expression in HCA-7 CRC cells. HCA-7 cells were seeded into 6-well plates (Nunclon delta, Fisher Scientific, Loughborough, LeicestershireUK) with Dulbecco’s modified Eagle’s medium (DMEM) (500 mL) in 10% foetal bovine serum (FBS) and incubated at 37 °C and 5% CO_2_. After 48 h, when the cells were almost 80% confluent, the CHS extracts were added and left for another 24 h The concentrations of the CHS used were based on their highest tolerated concentrations. Controls were also set up and these were: “no treatment” (HCA-7 cells in cell culture medium only); ethanol control (HCA-7 cells exposed to the equivalent volume of ethanol in the extracts, i.e., 0.2% *v/v*); and a positive control—HCA-7 cells exposed to a specific COX-2 inhibitor, Celecoxib (Sigma-Aldrich, Poole, Dorset, UK) (50 μM) [5,32], which was used at the highest concentration that could be tolerated by the cells without killing them. A positive control was used to gain some idea of the therapeutic potential of the CHS as Celecoxib has been shown to reduce adenomas in humans [33]. Celecoxib was made up in dimethyl sulfoxide (DMSO). After incubation with the CHS or the control, cells were lysed using LDS NUpage lysis buffer (Fisher Scientific, Loughborough, Leicestershire UK, 10718414) and then Western blotting was performed using equal amounts of sample (lysed cells) based on protein content, which was 30 μg. Following electrophoresis, the separated proteins were transferred on to Immobilon^®^ PVDF membranes (IPFL 00010; Merck Millipore, Watford, Hertfordshire, UK). Thereafter, the membrane was placed in blocking solution for at least 1 h and then primary antibodies were applied: COX-2(D5H5) XP^®^ Rabbit mAb #12282 (Cell Signalling, Leiden, Netherlands), (dilution 1:1000) and β-actin (1:1000; Cell Signalling, , Leiden, Netherlands) which was used as an internal control to show that equal amounts of protein were loaded. After incubating with the primary antibody, the membranes were washed with wash solution (5 min 3 times) and incubated with IRDye 689 Rd, donkey anti-Rabbit secondary antibody (LI-COR, Cambridge, UK). The signal was detected and quantified using LI-COR Image studio (LI-COR, Cambridge, UK). 

### 2.4. The Effect of Culinary Herb and Spice Extracts on COX-2 Activity, Based on PGE-2 Release, in HCA-7 CRC Cells 

The same CHS used in the COX-2 expression experiments were also used to investigate their effect on COX-2 activity in HCA-7 cells, which was determined by measuring their release of PGE-2. From the Western blot experiments, cell culture medium was collected and stored at −20 °C. Prior to carrying out the PGE-2 assay, samples were defrosted, centrifuged at 1000 rpm for 4 min and then assayed using a PGE-2 ELISA kit according to the manufacturer’s instructions (RND Systems, Abingdon, UK, KGE004B). To further investigate the effect of the CHS on COX-2 activity, the effect of the two most potent COX-2 CHS inhibitors, BE and TE, on COX-2 enzyme activity and PGE-2 production, in vitro, was investigated using COX-2 Inhibitor Screening Assay Kit (CAY560131-96; Cayman, Cambridge Bioscience, Cambridge, UK). 

### 2.5. The Effect of CHS Extracts on HCA-7 Cell Viability at 24, 48 and 72 h

The CHS found to have the strongest inhibitory effect on COX-2 expression were investigated to determine if and how they affected cell viability over the same time period (24 h) that their effect on COX-2 expression and activity was investigated. The CHS used were TE, GE, BLE, BLTE, and SGE. Cell growth was determined using the MTT (3-(4,5-Dimethylthiazol-2-yl)-2,5-Diphenyltetrazolium Bromide) assay: cells were trypsinised and seeded on 96-well plate and left for 24 h, thereafter the CHS extracts were added at concentrations based on the SRB investigation: 20, 10, 5, 2.5, 1.25, 0.625, 0.313, and 0.156 μg GAE/mL. Following the treatment periods (24 h as this was the period used for the COX-2 experiments, and also 48 and 72 h), the media containing the CHS extracts were removed and MTT (Sigma-Aldrich, Poole, Dorset, UK) (0.5 mg/mL) added. After 4 h, media containing MTT were removed and DMSO was added to solubilize the cells. Absorbances were then read at 570 nm (Epoch microplate reader, Biotek, UK) and the effect of the CHS on cell viability was determined and expressed in IC_50_ values. An additional experiment was performed to investigate what would happen if the CHS were removed after 24 h and replaced with fresh media and left for another 48 h. We hypothesised that, if after their removal, their IC_50_ values were similar to those obtained for the 72 h treatment, their action was cytotoxic. The lactate dehydrogenase (LDH) cytotoxic assay was also performed (Promega, Southampton, UK) to confirm cytotoxicity using the two most potent CHS extracts, BLE and TE, and their combination (BLTE). The latter was done to determine if the combination had a synergistic or antagonistic effect. The concentrations of the extracts were the same as for SRB and MTT assays and the treatment period was 72 h. The assay procedure was followed using the manufacture’s protocol.

### 2.6. The Effect of CHS on the Cell Cycle and Apoptosis in HCA-7 CRC Cells

Based on the results of the cell viability/cytotoxicity experiments, the most potent CHS extracts and their combinations were tested for their effect on cell cycle distribution (i.e., percentage of cells in sub G1, G1, S, and G2), and induction of apoptosis using FACS analysis. The CHS investigated were TE, GE, BLE, SE, RE, BLTE, BLSE, RTE, and SGE. Trypsinised cells (1 × 10^6^) were seeded into a flask containing 10 mL of cell culture medium and CHS extract. The doses used for the cell cycle analysis were based on the SRB growth inhibition study, and were slightly higher than their IC_50_ values (their approximate IC_70_) so that an effect could be observed without the CHS killing a large proportion of the cells. The exception was TE, for which a lower than IC_50_ dose was used because at the higher (approximate IC_70_) dose TE was killing most of the cells. Following the same exposure periods of 24 and 48 h used in the MTT cell viability experiments described above, supernatant and trypsinised cells were pooled together. Then cells were washed three times by centrifugation (at 1000 rpm for 4 min) and re-suspended in cold (4 °C) phosphate buffered saline (PBS). After the final wash, cells were re-suspended in 200 μL of cold PBS and fixed by adding 1 mL of ice cold 70% ethanol (in PBS). Cells were then kept overnight at 4 °C, and then washed 3 times as above. Thereafter, cells were incubated with 0.5 mL of propidium iodide (PI) buffer (BD Biosciences, Oxford, UK) for 30 min at room temperature and analysed using a FL3 detector (PI detector, 620 nm) (FACS calibur, BD Biosciences, Oxford, UK). At least 10,000 events were counted. Cells present in the sub G1 phase were considered to be apoptotic [34,35,36].

To confirm that apoptosis had occurred, a caspase-3/7 assay was performed using IncuCyte live-cell imaging according to the manufacturer’s instructions (EssenBioscience, Welwyn Garden City, Hertfordshire, UK). Briefly, cells were seeded on 96-well plates and placed into an incubator for 24 h, then one of the most potent extracts (BLE) was added at its approximate IC_70_ (for the reasons stated above) (6 μg GAE/mL) with the caspase-3/7 reagent. A caspase-3/7 inhibitor and Etoposide (Tocris Bioscience, Bristol, UK), a caspase activator, were used as a positive control for caspase 3 activation and a negative control, respectively. Another negative control (media without caspase-3/7 reagent) was set up to make sure cell culture medium did not generate a fluorescence signal. The untreated control contained just cell culture medium and caspase-3/7 reagent. On caspase-3/7 activation the probe emits a green fluorescent light which is detected by the IncuCyte camera. Cells were treated with the CHS extracts for 48 h and constantly monitored (images were taken every 2 h), and the data were analysed using IncuCyte ZOOM^®^ software (EssenBioscience, Welwyn Garden City, Hertfordshire, UK).

To further investigate the effect of the CHS on apoptosis, their (BLE and TE) effect on key protein markers of apoptosis were determined: cleaved caspase-3, p53, and cleaved PARP. Etoposide (25 μM) was used as a positive control for caspase-3 activation. All antibodies were purchased from Cell Signalling. The cell preparation and treatment were the same as for the COX-2 experiments.

### 2.7. Data Expression and Statistical Analysis

All experiments were done in triplicate (*n* = 3), which represents three separate experiments, and data are expressed as mean and standard error of the mean (±standard error mean (SEM)) unless otherwise stated. Growth inhibition data (SRB and MTT) are presented as 50% inhibitory concentration (IC_50_), the concentration at which 50% of cell growth is inhibited compared to the no treatment group (the control for which cell growth is 100%). The IC_50_ concentration was determined for each CHS (individual and in combination) extract (unless IC_50_ was not achieved) using Gen5 (Biotek, Swindon, UK) software and expressed as μg GAE/mL and DW equivalents μg/mL in order to show the importance of polyphenols found in the CHS extracts. To determine if synergy occurred as a result of the CHS combinations, the interaction factor (IF) was calculated for each combination using the analysis described by Gawlik-Dziki (2011). IF = IC_50_ value for combination/(IC_50_ value for herb1/2 + (IC_50_ value for herb2/2). IF values of <1 indicate synergy, IF values >1 indicate antagonism, and IF value of 1 indicate an additive effect.

Western blot band intensity was analysed using Odysey software (LI-COR, Cambridge, UK), the data were normalised against β-actin and any reduction in band intensity was expressed as a percentage in comparison with the intensity of the “no treatment” band (HCA-7 cells in cell culture medium only) which represented 100% expression.

COX-2 activity was determined based on PGE-2 release data, which are expressed as per cent reduction, in comparison to the control (HCA-7 cells in cell culture medium only), which represented 100% activity. One-way ANOVA with Tukey’s post-hoc test was performed to assess whether the differences in effect of the extracts were statistically significant. Pearson’s correlation coefficient (r) (2-tailed) was used to determine correlations between COX-2 expression, and PGE-2 production. To compare the IC_50_ values for the anti-proliferative, cell viability and cytotoxicity experiments, the independent sample test was performed. For all statistical tests, SPSS software was used and *p* < 0.05 was considered statistically significant. 

To determine if there was a statistically significant difference between treated (exposed to CHS) and untreated cells for the sub G1 phase, one-way ANOVA with Tukey’s post-hoc test was performed. 

## 3. Results

### 3.1. Effect of the CHS and Their Combinations on HCA-7 Cell Growth Using the SRB Assay

The CHS and their combinations were screened for anti-proliferative activity against the HCA-7 CRC cell line. TE (IC_50_: 3 ± 0.1 μg GAE/mL), BLE (4.7 ± 0.2 μg GAE/mL), and GE (5.5 ± 0.3 μg GAE/mL) were found to be the most effective extracts at inhibiting HCA-7 cell growth. For the combinations, BLTE produced the lowest IC_50_ value (3.3 ± 0.7 μg GAE/mL), followed by RTE (6 ± 0.4 μg GAE/mL) (Table 1). Treatment with a combination of CHS extracts was found to be synergistic in the majority of cases including SGE (IF = 0.67), SBLTE (IF = 0.80), and BLTE (IF = 0.90), and additive for RAE (IF = 0.98). In contrast, treatment with RTE was found to be antagonistic with (IF = 1.20) (Table 2). For the non-cancer cell line HFF-2, the extracts, specifically the most potent against HCA-7, BLE and TE, proved to be less potent based on their IC_50_s, which were 7.1 ± 0.6 μg GAE/mL and 7.1 ± 0.9 μg GAE/mL, respectively.

### 3.2. The Effect of CHS Extracts on COX-2 Expression in HCA-7 CRC Cells

**Figure 1:**
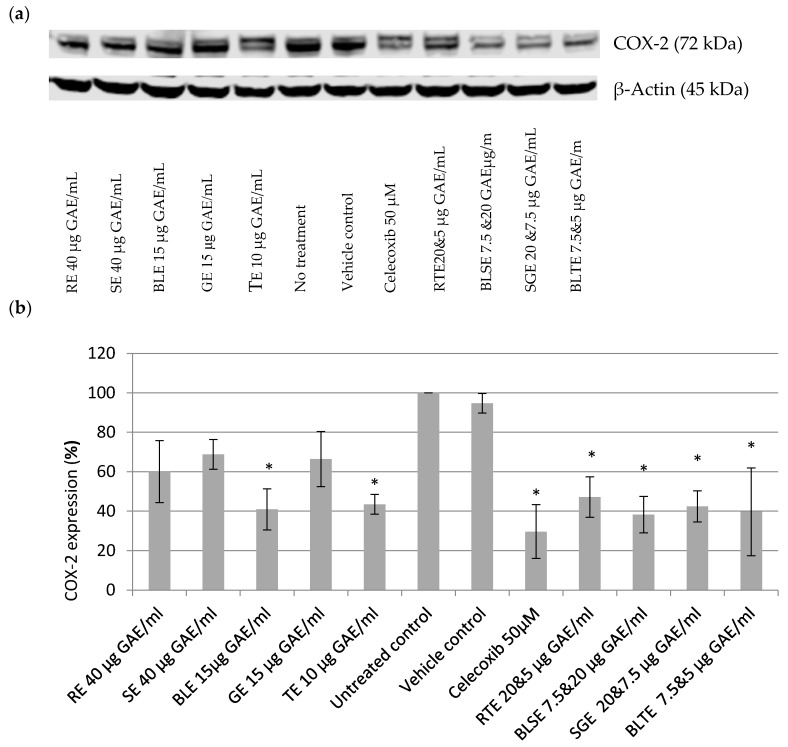
Effect of CHS extracts on on COX-2 expression: (**a**) Western blot; and (**b**) quantitative analysis of COX-2 bands. Data are expressed in comparison to control (100%) after the signal was normalized against β-actin. rosemary ethanol (RE); sage ethanol (SE); bay leaf ethanol (BLE), ginger ethanol (GE), and turmeric ethanol (TE) and their combinations (rosemary and turmeric ethanol (RTE), bay leaf and sage ethanol (BLSE), sage and ginger ethanol (SGE) on COX-2 expression in HCA-7 cells. * Statistically significant different from control (*p* < 0.05), *n* = 3, ±SEM. Untreated control contained just DMEM with 10% FBS (vehicle control–ethanol was 0.4% (*v*/*v*), the highest amount found in the extracts).

Based on the SRB results, the most potent extracts were used for the COX-2 experiments. As shown in Figure 1, treatment with individual extracts RE, SE, BLE, GE, and TE resulted in the reduction of COX-2 expression in HCA-7 cells (Figure 1a,b). The strongest effect was seen with the highest tested concentrations that were tolerated by the cells (i.e., 40 μg GAE/mL for RE and SE; 15 μg GAE/mL for GE and BLE, and 10 μg GAE/mL for TE). BLE and TE extracts were the most potent; they reduced COX-2 expression by 59% and 57% respectively. The four combinations used, RTE, BLTE, SGE, and SBLE, reduced COX-2 expression by 53%, 60%, 58% and 62%, respectively. The effects of the most potent of the CHS extracts and combinations were slightly less but of the same magnitude as that of the selective COX-2 inhibitor Celecoxib (50 μM), which reduced COX-2 expression by 70%.

### 3.3. The Effect of Culinary Herb and Spice Extracts on COX-2 Activity, Based on PGE-2 Release, in HCA-7 CRC Cells 

**Figure 2:**
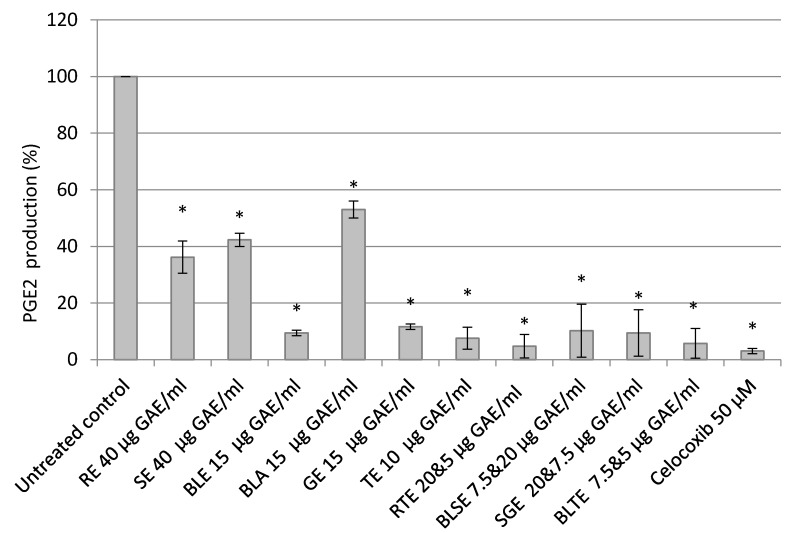
Effect of CHS (RE, SE, BLE, GE, and TE) and their combinations (RTE, BLSE, SGE, and BLTE) on PGE-2 release from HCA-7 cells. * Untreated control contained just DMEM with 10% FBS (vehicle control–ethanol was 0.4% (*v*/*v*), the highest amount found in the extracts). Rosemary ethanol (RE), sage ethanol (SE), bay leaf ethanol (BLE), ginger ethanol (GE), turmeric ethanol (TE), and rosemary and turmeric ethanol (RTE), bay leaf and sage ethanol (BLSE), sage and ginger ethanol (SGE), bay leaf and turmeric ethanol (BLTE). * Indicates statistically significant difference from control (*p* < 0.05), *n* = 3, ±SEM.

Treatment with the CHS extracts resulted in significant inhibition of PGE-2 release with TE (92%), BLE (91%), and GE (88%) almost completely inhibiting its release. Of the combinations, BLTE (92%) and RTE (91%) had the strongest inhibitory effect (Figure 2). Celecoxib (50 μM) reduced the PGE-2 release by 97% (Figure 2). Furthermore, most extracts produced a stronger reduction in PGE-2 release than COX-2 expression. There was a strong (*r* = 0.78) statistically significant (*p* < 0.05) correlation between PGE-2 release and COX-2 expression. 

**Figure 3:**
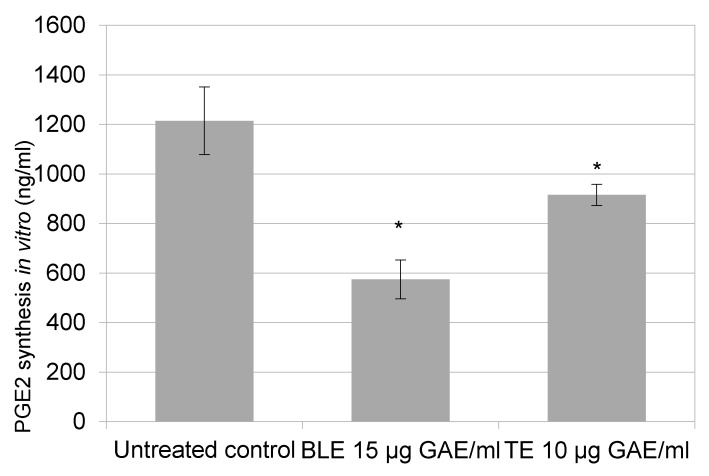
BLE and TE effect on COX-2 activity and PGE-2 production. Untreated control contained the same volume of ethanol that was found in extracts to ensure that the final reaction volume would be the same. DW equivalents for BLE (bay leaf in ethanol) 15 μg GAE/mL = 566 μg/mL and for TE (turmeric in ethanol) 10 μg GAE/mL = 1000 μg/mL. * Indicates statistically significant difference from control (*p* < 0.05), *n* = 3, ±SEM.

To confirm that BLE and TE directly targets COX-2 activity, rather than just purely reducing its expression, and that the effect observed was not due to the inhibition of HCA-7 cell growth by the CHS, an in vitro COX-2 inhibition screening assay was performed. The assay revealed that BLE and TE reduced PGE-2 production by 53% and 25%, respectively (Figure 3).

### 3.4. The Effect of CHS Extracts on HCA-7 Cell Viability at 24, 48, and 72 h

**Figure 4:**
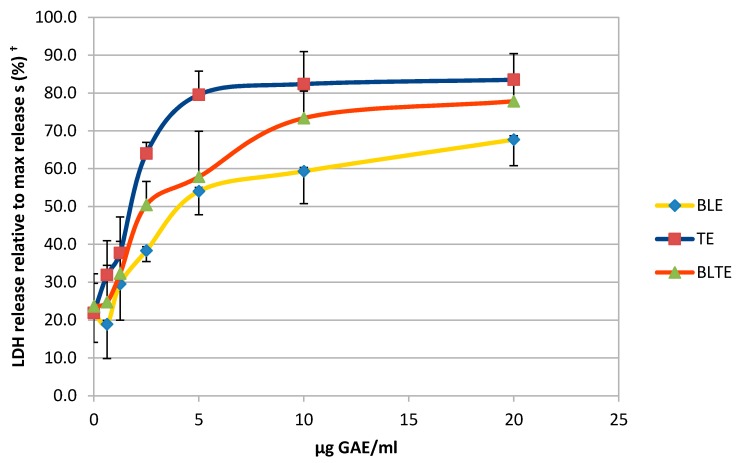
Cytotoxic effect of CHS against HCA-7 cells using LDH assay. BLE (bay leaf in ethanol), TE (turmeric in ethanol), BLTE (bay leaf and turmeric in ethanol). ^†^ Data are expressed as a percentage of maximum release of lactate dehydrogenase (LDH), *n* = 3.

The CHS extracts that possessed the strongest COX-2 inhibitory activity were then used to determine their effect on cell viability over the same period used for the COX-2 experiments. TE had the lowest IC_50_ values across all three time points (24, 48, and 72 h): 6 ± 0.1, 2.1 ± 0.5, and 2.5 ± 0.3 μg GAE/mL, respectively (Table 3). At the 24 h time point, for all extracts and combinations, the IC_50_ values were higher than those for the 48 and 72-h treatments. The impact of removing the treatment and replacing it with fresh medium was also investigated. Results revealed that the removal of extracts after 24 h did not have a significant effect on the IC_50_ values, in comparison to the whole 72-h treatment, the difference between the IC_50_ values were statistically insignificant (Table 3), suggesting that the effect of the CHS extracts is cytotoxic. To confirm their cytotoxic action, a LDH assay was performed using the most potent CHS extracts, TE and BLE, and results clearly indicate that at the higher concentration (5, 10, and 20 μg GAE/mL) both produced cytotoxic effects (Figure 4).

### 3.5. The Effect of CHS on the Cell Cycle and Apoptosis in HCA-7 CRC Cells 

**Figure 5:**
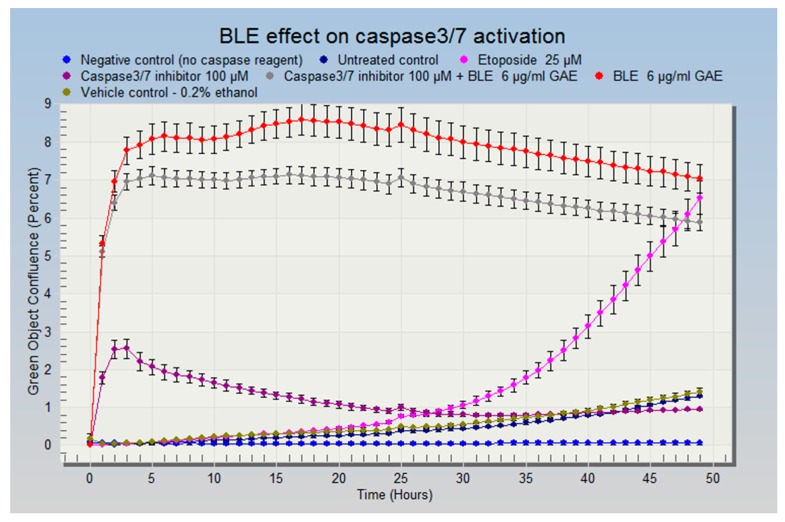
BLE effect on cell death and caspase-3/7 activation.

**Figure 6:**
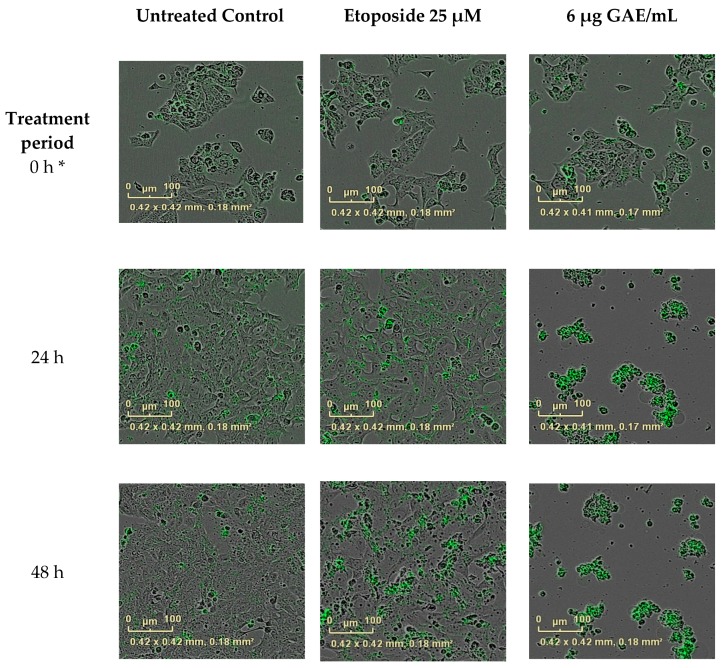
BLE effect on caspase activation. Images recorded using the IncuCyte ZOOM^®^ camera (×10 zoom). HCA-7 cells were treated with bay leaf ethanol (BLE) at 6 μg GAE/mL concentration. * The first scan taken by the IncuCyte ZOOM^®^; before the first scan was performed cells were exposed to the treatment for ~30 min.

**Figure 7:**
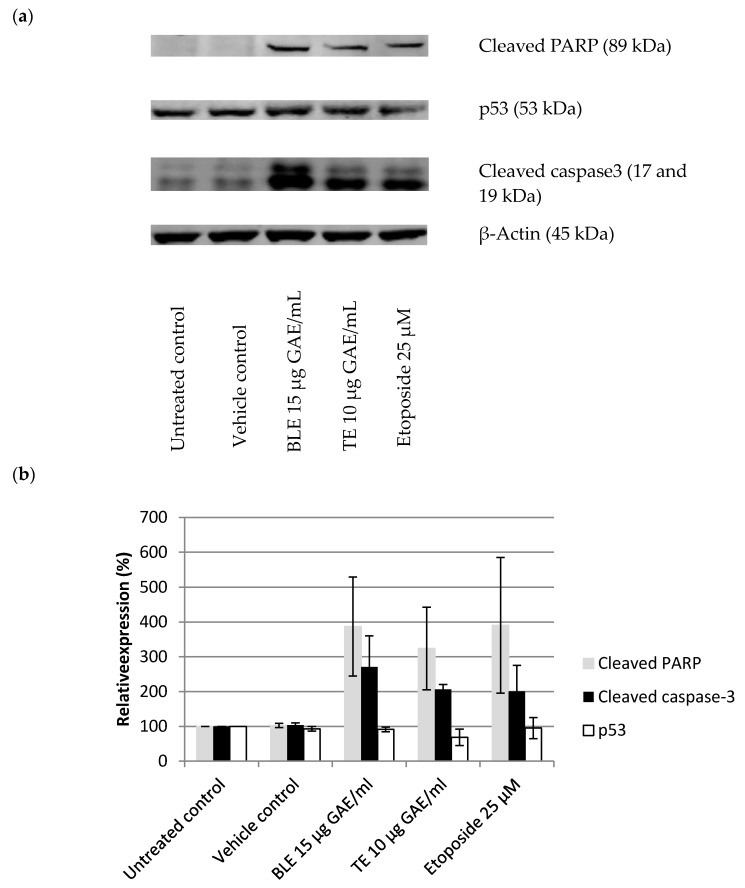
BLE and TE effect on proteins markers for apoptosis in HCA-7 cell line. (**a**) Western blot” Cells were treated for 24 h with bay leaf (BLE 15 μg GAE/mL), turmeric (TE 10 μg GAE/mL), and Etoposide 25 μM, which was used as a positive control for caspase-3 activation. (**a**) Quantitative analysis of Western blot bands. Protein expression was normalised against β-Actin and expressed relative to untreated control, where control is 100%; (**b**) Untreated control contained just DMEM with 10% FBS (vehicle control–ethanol was 0.4% (*v*/*v*), the highest amount found in the extracts). Bay leaf in ethanol (BLE), turmeric in ethanol (TE), *n* = 3, ±SEM.

In light of the results of the growth inhibition experiments, the most potent CHS extracts and their combinations were tested for their effect on HCA-7 cell cycle regulation 24 h post treatment. As shown in Table 4, these treatments resulted in an increased number of cells undergoing apoptosis due to their accumulation in the sub G1 phase of the cell cycle (Table 4). BLE and GE were the most potent extracts, causing 28% and 27%, respectively, of the cells to accumulate in the sub G1 phase. Of the combinations, treatment with BLTE induced the greatest percentage of cells (33%) being accumulated in the sub G1 phase 24 h post treatment. These effects were more pronounced when the cell cycle analysis was conducted 48 h post treatment (Table 4). Treatment with BLTE and RTE combinations resulted into 35% and 33% of cells entering the sub G1 of the cell cycle. BLTE treatment was also accompanied by a reduction in the number of cells in the S and G2 phases of the cell cycle. To confirm apoptosis, a caspase-3/7 assay was performed with one of the most potent extracts, BLE. The results showed that BLE extract (6 μg GAE/mL) activated caspase-3/7 (Figure 5 and Figure 6) and that the activation of caspase-3/7 by BLE was not inhibited by the presence of the caspase-3/7 inhibitor. Finally, the effects of the most potent extracts (BLE and TE) on proteins markers for apoptosis (i.e., cleaved PARP and cleaved caspase 3) were investigated to ascertain further how apoptosis was induced by these CHS. Both extracts increased the expression of cleaved caspase-3, and cleaved PARP and the increase was comparable to that of the caspase 3 activator - Etoposide. In contrast, BLE did not affect the expression of p53 whilst its expression was reduced slightly following treatment with TE (Figure 7a,b). 

HCA-7 cells were treated with BLE (6 μg GAE/mL); Etoposide (25 μM) was used as a positive control for caspase-3 activation, caspase-3/7 inhibitor (100 μM) was used as a negative control. Another negative control—(media without caspase-3/7 reagent) was used to ensure the cell culture media does not generate fluorescence signal. Vehicle control—0.2% ethanol *(v/v*). Before the first scan was performed by the the IncuCyte ZOOM^®^, cells were exposed to the treatment for ~30 min so time 0 is approximately 30 min after cells were exposed. On caspase-3/7 activation, the reagent turned green and was recorded by IncuCyte ZOOM^®^ camera.

## 4. Discussion

The aim of the present study was to investigate the effect of a selection of CHS (individually and in combination) on COX-2 expression and activity in HCA-7 CRC cells and to establish if this activity is linked to any inhibition by the CHS of the growth of these cells.

Some research suggests that whole food extracts can be more effective than isolated compounds [37,38,39], and the present study clearly demonstrates the potential benefits of CHS, individually and in combination, in relation to CRC. The IC_50_s established using the SRB and MTT assays as GAE and DW equivalents clearly show that the action of these CHS is likely due to their polyphenolic constituents and not the amount of the CHS used, as their potency in IC_50_ expressed as μg GAE/mL differs from those expressed as per gram of dry weight. In addition, bearing in mind that the polyphenol profiles of the CHS differ, the data suggest that specific polyphenols in the CHS influence the effect on CRC cell growth. 

The growth inhibition results are in line with other studies that demonstrated that CHS possess anti-proliferative activity against various cancer cell lines including CRC [22,23,36,40,41,42]. However, to our knowledge, none of the above-cited studies investigated the effect of the CHS used in the present study (individually or in combination) on the HCA-7 CRC cell line. The SRB growth inhibition results also suggest that the action of the CHS may be cancer cell specific as they, specifically BLE and TE, had a far less potent effect when exposed to the HFF-2 fibroblast cells. These data are supported by growth experiments using Incucyte which show that the doses of BLE and TE required to inhibit HCA-7 cell growth were lower than those required to inhibit the growth of the HFF-2 cells (see Supplementary Materials, Figures S1 and S2). 

The majority of tested CHS extracts clearly inhibited the growth and reduced viability of HCA-7cells, as shown using the SRB, MTT, and LDH assays, (Table 1 and Table 2). Interestingly, for the same extracts, the IC_50_ values varied when determined using the SRB and MTT assays. The differences in the IC_50_ values could be explained by the different mechanisms upon which these assays are based: MTT utilises NAD(P)H-dependent cellular oxidoreductase enzymes that convert colourless tetra-zolium to the purple-coloured formazan dye, whilst SRB measures cell mass and does not distinguish between dead and live cells [43]. In addition, it must also be noted that for the MTT experiments the exposure times were shorter than that for the SRB assay. Nevertheless, TE, GE, and BLE were the most potent extracts based on both assays.

Combinations of the CHS were also investigated to determine if combining them had an additive, synergistic or possibly even an antagonistic effect on cell growth. Based on the results of the SRB assay, the most potent combination (BLTE) included two of the most potent individual CHS constituent extracts (TE and BLE) (based on the SRB and MTT assays (Table 1 and Table 2)). The combinations that included TE, BLTE, and RTE, were less potent than TE, which was the strongest individual CHS extract. In fact, based on the IC_50_ values alone, none of the combinations were more potent than both their constituent CHS, suggesting that their effects were neither additive nor synergistic. However, based on the IF index, there is evidence of both additive and synergistic effects, which is in keeping with research on the effect of combinations of culinary and medicinal herbs on CRC cell growth, namely that by Yi and Wetzstein [23]. The IF data also provide evidence of antagonism (RTE; Table 2) despite the fact that this combination proved to be quite a potent inhibitor of HCA-7 cell growth as indicated by its IC_50_ (Table 1). It is clear that combining the CHS resulted in a complex set of phytochemical interactions, which regardless of their effect, be it additive, synergistic or antagonistic, resulted in the inhibition of HCA-7 cell growth. The overall potency of these combinations does indicate that they possess some chemotherapeutic potential. One limitation of this part of the study is that only one ratio (1:1) was used for the combinations, and from it the interaction factor (IF) was calculated. Although this factor is a quick method for identifying synergistic, antagonistic and additive effects of the combinations used, to obtain a fuller picture of the nature of these interactions the effect of different ratios of the same combinations needs to be investigated [44,45]. 

Based on the SRB results, the effect of the most potent CHS on COX-2 expression was investigated. The present study showed that certain CHS decreased COX-2 expression at the protein level and also inhibited the synthesis, and consequently the release, of PGE-2. The inhibitory effect on PGE-2, could in part be due to a reduction in cell number caused by the treatment with CHS, however, a follow up experiment showed that the CHS, specifically BLE and TE, directly inhibit the activity of COX-2 and the synthesis of PGE-2, indicating that their effect is due to both inhibition of the expression and activity of COX-2 (Figure 1, Figure 2 and Figure 3). One of the striking observations of this part of the present investigation was the effect of a number of the CHS (individual and in combination) on COX-2 expression and activity (specifically PGE-2 release) in comparison to that of the COX-2 specific inhibitor Celecoxib, which is an established treatment for a number of conditions [46]. The effect of some of the CHS was comparable to that of Celecoxib, supporting their not inconsiderable potency, which is likely due primarily to their polyphenol content. This is a key point to note in light of literature suggesting that in comparison to anti-inflammatory drugs including Celecoxib, food polyphenols are of limited biological relevance regarding their effect on COX-2 activity [47]. Although Willenberg et al. [47] investigated the effects of food polyphenols that are not major constituents of the CHS used in the present study, their focus on the individual constituents rather than their food sources may explain the lack of potency and reinforces the need to consider these constituents within their food matrices in which interactions likely influence the biological potency of the whole food. 

The individual CHS that proved to be the most potent in inhibiting COX-2 expression and activity (PGE-2 release and synthesis) were TE and BLE (Figure 1 and Figure 2). Both extracts were also among the most potent at inhibiting HCA-7 cell growth and reducing its viability, suggesting that there is a link between downregulation of COX-2 expression/activity and growth inhibition. Such an association is supported by the work of Levi-Ari et al [47], who found that the growth inhibitory IC_50_ values of curcumin were lower for a COX-2 positive cell line (HT-29; 15 μM) than SW480 (40 μM), which does not expresses COX-2. Studies on the anti-inflammatory properties of individual food polyphenols indicate that the inhibitory effect of these CHS on COX-2 is via their polyphenolic constituents. However, it must be borne in mind that a number of factors including the phenolic composition of the CHS, which varies depending on solvents used, and interactions between constituents likely affect their anti-inflammatory and other bioactive properties [38,48,49,50,51]. 

Research has clearly established the anti-inflammatory effects of turmeric on non CRC cells are primarily due to the action of its major bioactive polyphenolic constituent curcumin [26]. Curcumin has been shown to possess a broad anti-carcinogenic activity by targeting various pro- and anti-carcinogenic pathways [52]. Indeed, Zhang et al. [24] demonstrated that curcumin (10–20 μM) blocked the induction of COX-2 expression by bile and the phorbol ester (phorbol-12-myristate-13-acetate (PMA)) in HCA-7 cells and other gastrointestinal cancer cell lines. Moreover, in the same study, curcumin also suppressed PGE production. Another study found that curcumin at low concentrations (5–75 μM) reduced COX-2 expression in HT29 cells (CRC) [26]. It is unclear how curcumin, and thus turmeric, act to inhibit COX-2 activity. One possible way in which this polyphenolic constituent affects COX-2 expression is by targeting the transcription factor NF-κB, which is involved in regulating COX-2 expression [53,54]. Another is via its action on activator protein-1 (AP-1), which is a downstream transcription factor that regulates COX-2 expression [25,55]. Furthermore, COX-2 is a bifunctional enzyme with cyclooxygenase and peroxidase activities. First, cyclooxygenase converts arachidonic acid to prostaglandin G2, which then is converted to prostaglandin H2 (PGH2) by the peroxidase enzyme, and then specific enzymes convert PGH2 to prostaglandin E2 (PGE-2) and other prostaglandins [56,57]. The bifunctional property of COX-2 may also be a target as curcumin inhibits both cyclooxygenase and peroxidase activities [25], which is an additional way through which curcumin, and thus turmeric, could reduce the level of PGE-2, and potentially be more advantageous in chemoprevention than non-steroidal anti-inflammatory drugs that only target cyclooxygenase and have no effect on the peroxidase [25]. Curcumin (~1 μM) has also been shown to decrease PGE-2 synthesis by inhibiting microsomal PGE-2 synthase-1 activity, which is functionally linked to COX-2 and is required to convert PGH2 into PGE-2 [58]. However, other CHS polyphenols, namely rosmarinic acid and [6]-gingerol, showed inhibitory activity against microsomal PGE-2 synthase-1 [58], which suggests that a very specific structure of a polyphenol is needed to target this enzyme. 

Curcumin may not be the only bioactive compound in turmeric that is responsible for the effects the authors observed in the present study as other constituents (turmerones, elemes, furanodiene, cyclocurcumin, bisacurone, and germacrone) of this spice have recently been identified as possessing anti-inflammatory activity and targeting various pro-inflammatory molecules including COX-2, PGE-2, and NF-κB [59]. Thus, as alluded to above, the effect of turmeric on COX-2 and PGE-2 may also be due to the combined effect of a number of its phytochemical constituents.

Regarding bay leaf and ginger, it would not be unreasonable to assume that their polyphenolic constituents also contributed to their COX-2/PGE-2 inhibitory action. In the present study, bay leaf, specifically BLE, proved to be almost as potent as that of TE. Bay leaf is a less studied herb, a small number of studies have reported its ability to decrease COX-2 expression (in macrophages) [60,61] and also to moderately inhibit COX-2 activity [42]. However, in the latter study, processed bay leaf (cooked and enzymatically treated) was used and the inhibition of cellular COX-2 expression and activity was not investigated. Willenberg et al. [46] found that naringenin and apigenin, which are present in this herb, reduced COX-2 expression and activity in HCA-7 cells. However, these polyphenols are only present in bay leaf in trace amounts [62,63] and hence are unlikely to be the main polyphenols responsible for the significant reduction in COX-2 expression by BLE. Other potential constituents (not all phenolic) that may contribute to the anti-inflammatory activity of bay leaf are dehydrocostus lacton, limonene, β-sitosterol, eugenol, p-coumaric acid, ferrulic acid, and eremanthin. However, their presence and amount can vary depending on the solvent used for the purposes of extraction [63,64]. Ginger (GE) also reduced COX-2 expression in the present study (Figure 1 and Figure 2). However, the results suggest that its effect on activity was greater than on expression. Ginger extract has been shown to reduce COX-2 gene expression in another COX-2 expressing CRC cell line—HT29 [65] and studies indicate that it is its main active phenolic constituents, gingerols, shogaol, and paradols that possess anti-inflammatory and anti-carcinogenic properties against a range of cancer, including CRC, cells [66,67,68]. Indeed, it has been shown that gingerols and shagols inhibited COX-2 activity by selectively binding to this enzyme with high affinity [69]. It is therefore possible that in the present study the action of the other CHS on COX-2 activity may be due to potent polyphenolic inhibitors.

The effect of combinations of the CHS on COX-2 expression and activity suggests that some additive or possibly synergistic effects came into play as some of the combinations, specifically RTE, BLTE, and BLSE produced slightly stronger effects than those of their constituent individual CHS extracts (Figure 1 and Figure 2). Both BLTE and BLSE had synergistic effects on HCA-7 cell growth based on their respective Ifs, strengthening the possibility of a link between the inhibition of COX-2 and HCA-7 cell growth by the CHS. However, for RTE, its effect on HCA-7 cell growth was, based on its IF, shown to be antagonistic (Table 2). Such results highlight further the complexity of how whole foods in combination work as bioactive agents. Numerous factors come into play, which have already been touched on above and include the constituent CHS used, and the ratios of the constituents in the combinations. There is evidence in the literature that combining several foods can result in synergistic effects, suggesting that some combinations are more beneficial than the constituent single food [70]. However, the literature also reports that food combinations can also give rise to antagonistic effects [71], although this was not the case in the present study, thus highlighting the complexity of the “within matrix” interactions. It is clear that as with the growth inhibition results discussed above, the effect of such combinations require further investigation. 

The results of the present study clearly show that a selection of CHS (individually and in combination) inhibit the growth of the HCA-7 cell line and its COX-2 expression and activity (Figure 1, Figure 2 and Figure 3). It is well established that COX-2, and its product PGE-2, play an important role in carcinogenesis of CRC [5], including initiation of cancer cell growth, promoting proliferation, survival, angiogenesis, and migration, by creating a tumour-favourable microenvironment and stimulating metastasis. Furthermore, it has been shown that PGE-2 suppresses apoptosis and stimulates cancer cell proliferation [65,72]. The present study also showed, using the most potent COX-2/PGE-2 CHS inhibitors—BLE, BLTE, GE, TE, and SGE—that they were cytotoxic, and not cytostatic, within the same time frame that they were shown to inhibit COX-2 (Table 3), indicating that they acted by killing the cells rather than halting their proliferation. The subsequent cell cycle and apoptosis experiments, using BLE and TE, indicate that these extracts are capable of inducing apoptosis through the increase in the number of cells in the sub G1 phase, which was irreversible as confirmed by the activation of the apoptotic effector caspases-3 and 7 (Table 4, Figure 5 and Figure 6). Their pro-apoptotic action (Figure 7) was further confirmed by the increase in cleaved caspase 3 and cleaved PARP. The effect on the former further indicates the growth inhibition, via apoptosis, was irreversible and was comparable to that of the caspase activator Etoposide [73]. PARP protein is considered a hallmark of the apoptosis process and it is targeted by caspase-3 [74,75]. The results of the present study further strengthen the evidence that these extracts can target the pathways involving caspase-3 and cleaved PARP. Dimas et al. [36] reported that whole turmeric ethanol extract increased the number of CRC cells (HCT116) in sub G1 phase, whilst there were no changes in other cell cycle phases. In addition, studies have shown that curcumin is capable of activating caspase-3 [76], and increasing cleaved PARP [77]. These findings are very similar to those of the present study. There is relatively little known about the effect of bay leaf on the cell cycle and apoptosis in CRC cells with the exception of, to the authors’ knowledge, the work of Rodd et al. [78], who reported that unfractionated and low molecular mass fractions of bay leaf were pro-apoptotic (based on caspase-3/7 activity) although their impact varied. In addition, they halted the cell cycle via arrest in the G1/S phase.

Regarding p53, the slight reduction in pro-apoptotic p53 caused by TE was unexpected. However, the HCA-7 cell line has a partially mutated/dysfunctional p53, which in fact can suppress apoptosis [78,79]. Thus, the most likely explanation therefore is that TE simply reduced mutated p53. Indeed, it has been shown that turmeric and curcumin are able to degrade mutated p53 in skin cancer cells [80]. Another study demonstrated that curcumin reduced levels of p53 expression in CRC cells (HCT15) [76]. In the present study, bay leaf (BLE) appeared to have little effect on the expression of p53, suggesting that its apoptotic action does not involve this protein, although further studies are required to confirm this observation. 

The results of the cell cycle/apoptosis analyses in the present study suggest that there is an association between COX-2 inhibition and apoptosis by the CHS in HCA-7 CRC cells. This suggestion is supported by Aggarwal et al. [81], although the focus of their study was curcumin rather than a selection of CHS. The same study showed that curcumin induced apoptosis in HCT116, a CRC cell line that does not express COX-2, although the effect was slightly lower. Therefore, the apoptotic action of the CHS in CRC cells may involve COX-2 dependent/independent inhibition, and the type of inhibition may influence their potency. 

## 5. Conclusions

It is clear from the present study that the investigated CHS, both individual and in combination, significantly inhibited the growth, via apoptosis, of HCA-7 CRC cells within the same time frame as their inhibition of COX-2 expression and activity. The levels of COX-2 inhibition were similar to those achieved by Celecoxib, which is a strong selective COX-2 inhibitor, highlighting the possible therapeutic potential of these foods. Our findings also suggest that combining several CHS could produce beneficial growth inhibitory and anti-inflammatory effects. However, further work is required to ascertain whether or not the growth inhibitory effect of the CHS is influenced by COX-2 inhibition. In addition, in light of the effect of the CHS in comparison to Celecoxib and Etoposide, determination of their therapeutic potential, both individually and in combination, on a larger panel of CRC cells is required. 

## Figures and Tables

**Table 1:** The effect of CHS and their combinations on HCA-7 cell growth using the SRB assay.

Herb/Spice/Combinations	IC_50_ (μg GAE/mL)	IC_50_ (μg/mL of DW)
TE	3.0 (±0.3)	300
BLTE	3.3 (±0.7)	227
BLE	4.7 (±0.2)	177
GE	5.5 (±0.3)	417
BLSE	5.5 (±0.3)	180
RTE	6.0 (±0.4)	382
SGE	6.8 (±0.1)	352
SE	12.5 (±0.9)	347
SAE	15.7 (±0.6)	414
RE	15.9 (±0.4)	347
RAE	16.2 (±0.4)	432
RA	17.1 (±0.1)	442
SA	>20 (n/a)	>442

GAE: gallic acid equivalent, used to express the total polyphenol content. The IC_50_ values are also expressed in dry weight (DW) equivalent of the herb/spice. Culinary herbs and spices (CHS), sulforhodamine B (SRB), Turmeric in ethanol (TE), ginger in ethanol (GE), bay leaf in ethanol (BLE), sage in ethanol (SE), sage in water (SA), rosemary in ethanol (RE) and rosemary in water (RA), rosemary in water and rosemary ethanol (RAE), sage in water and sage ethanol (SAE), bay leaf and turmeric ethanol (BLTE), sage and ginger ethanol (SGE), bay leaf and sage ethanol (BLSE), and rosemary and turmeric ethanol (RTE). Each value is the values are expressed as mean of triplicate samples, ±SEM, *n* = 3.

**Table 2:** IF * index for CHS extract combinations based on SRB assay.

Combinations	HCA-7
RAE	0.98
SAE	n/a
RTE	1.20
BLTE	0.90
SGE	0.67
SBLE	0.80

Bay leaf and turmeric ethanol (BLTE), sage and bay leaf ethanol (SBLE), rosemary aqueous ethanol (RAE), sage aqueous and ethanol (SAE), and sage and ginger ethanol (SGE). * The IF value for SAE could not be calculated because the IC_50_ value for SA was not achieved.

**Table 3:** Effect of CHS and their combinations on HCA-7 cell viability using MTT assay.

Herbs/Spices	24 H	48 H	72 H	Extracts Removed from Media *
	IC_50_ (μg GAE/mL) (±SEM)	IC_50_ (μg GAE/mL) (±SEM)	IC_50_ (μg GAE/mL) (±SEM)	IC_50_ (μg GAE/mL) (±SEM)
TE	6.0 (±0.1)	2.1 (±0.3)	2.5 (±0.2)	2.5 (±0.4)
GE	10.0 (±0.5)	6.1 (±0.6)	5.8 (±0.1)	7.8 (±0.8)
BLE	10.5 (±0.3)	6.0 (±0.5)	9.2 (±0.2)	8.4 (±0.6)
BLTE	11.1 (±0.9)	4.9 (±0.5)	3.6 (±0.6)	3.7 (±0.6)
SGE	11.1 (±0.8)	10.7 (±0.5)	10.9 (±0.8)	11.3 (±0.9)

* Extracts removed after 24 h and replaced with fresh media and left for another 48 h. Difference between the IC_50_ values were statistically insignificant compared to 72 h treatment *p* > 0.05; *n* = 3. GAE: gallic acid equivalent, which is used to express the total polyphenol content. Turmeric ethanol (TE), ginger ethanol (GE), bay leaf ethanol (BLE), bay leaf and turmeric ethanol (BLTE), sage and ginger ethanol (SGE).

**Table 4:** The Effect of CHS on the cell cycle in HCA-7 cells over 24 and 48 h.

Herbs/Spices	Sub G1 (%) (±SEM)		G1 (%) (±SEM)		S (%) (±SEM)		G2 (%) (±SEM)	
	24 h	48 h	24 h	48 h	24 h	48 h	24 h	48 h
Untreated control	10 (±1.3)	7 (±1.4)	40 (±1.5)	46 (±1.0)	26 (±1.2)	25 (±2.3)	23 (±1.2)	20 (±2.9)
Vehicle control (ethanol)	9 (±1.2)	4 (±0.7)	41 (±1.6)	47 (±1.3)	27 (±1.2)	24 (±1.2)	23 (±0.4)	23 (±0.3)
Vehicle control (H_2_O)	10 (±1.0)	4 (±1.0)	39 (±0.9)	45 (±0.6)	25 (±1.8)	25 (±0.3)	24 (±1.2)	23 (±0.3)
TE (2 μg GAE/mL)	23 (±4.0) *	49 (±3.1) *	41 (±3.2)	21 (±4.7)	20 (±0.3)	19 (±1.2)	15 (±0.9)	9 (±0.6)
GE (8 μg GAE/mL)	27 (±3.9) *	49 (±3.1) *	41 (±2.3)	25 (±2.9)	17 (±0.3)	15 (±1.2)	12 (±0.6)	8 (±0.7)
BLE (6 μg GAE/mL)	28 (±3.2) *	43 (±2.5) *	38 (±3.4)	28 (±1.5)	20 (±1.8)	15 (±1.2)	13 (±1.8)	12 (±1.0)
SE 16 μg GAE/mL	16 (±2.3)	30 (±1.3) *	42 (±0.3)	31 (±4.2)	23 (±1.2)	21 (±3.5)	18 (±1.5)	17 (±0.3)
RE (20 μg GAE/mL)	14 (±0.3)	17 (±4.3)	42 (±0.9)	41 (±1.2)	31 (±0.7)	20 (±0.4)	10 (±0.3)	20 (±1.6)
BLTE (3 μg GAE/mL BL and 1 μg GAE/mL TE)	33 (±0.9) *	33 (±0.6) *	34 (±0.3)	35 (±1.3)	19 (±0.6)	16 (±0.7)	11 (±0.7)	14 (±1.5)
BLSE (3 μg GAE/mL BL and 8 μg GAE/mL SE)	19 (±0.8) *	26 (±1.8) *	41 (±0.8)	42 (±3.2)	18 (±1.6)	18 (±1.7)	12 (±0.4)	14 (±1.0)
RTE (10 μg GAE/mL RE and 1 μg GAE/mL TE)	16 (±0.9) *	35 (±0.3) *	42 (±1.5)	32 (±2.5)	26 (±2.3)	17 (±1.2)	18 (±0.3)	12 (±0.3
SGE (8 μg GAE/mL SE and 4 μg GAE/mL GE)	23 (±0.3) *	22 (±1.2)	37 (±0.7)	45 (±0.7)	27 (±0.3)	20 (±0.6)	11 (±0.6)	12 (±1.0)
Celecoxib (50 μM)	23	-	45	-	18	-	13	-

Data are expressed as a percentage of cells in each phase (*n* = 3). * Statistically significant difference in comparison to control (*p* < 0.05). Vehicle control (ethanol)—0.2% (*v*/*v*), the highest volume found in the extracts. Vehicle control (filtered, sterilised and distilled H_2_O) 0.7% (*v*/*v*), the highest volume found in the extracts. Rosemary ethanol (RE), sage ethanol (SE), bay leaf ethanol (BLE), ginger ethanol (GE), turmeric ethanol (TE), and rosemary and turmeric ethanol (RTE), bay leaf and sage ethanol (BLSE), sage and ginger ethanol (SGE), bay leaf and turmeric ethanol (BLTE).

## Supplementary Materials

The following are available online at www.mdpi.com/2072-6643/9/10/1051/s1, Figure S1: (**a**) BLE effect on HFF-2 cell growth, (**b**) TE effect on HFF-2 cell growth, Figure S2: (**a**) BLE effect on HCA-7 cell growth, (**b**) TE effect on HCA-7 cell growth.

## Abbreviations

The following abbreviations are used in this manuscript:

A	aqueous
BLE	bay leaf ethanol
BLTE	bay leaf ethanol and turmeric ethanol
COX-2	Cyclo-ooxygenase 2
DMEM	Dulbecco’s modified Eagle’s medium
E	ethanol
GAE	gallic acid equivalent
GE	ginger ethanol
PGE-2	prostaglandin E2
RA	rosemary aqueous
RE	rosemary ethanol
RTE	rosemary ethanol and turmeric ethanol
SA	sag aqueous
SBLE	sage ethanol and bay leaf ethanol
SE	sage ethanol
SGE	sage ethanol and ginger ethanol
