# Supplementary material for: Inhibitory Effects of Culinary Herbs and Spices on the Growth of HCA-7 Colorectal Cancer Cells and Their COX-2 Expression
Source: Nutrients. 2017 Sep 21;9(10):1051. doi: 10.3390/nu9101051 (PMC5691668; doi:10.3390/nu9101051)
Supplement: Supplementary file 1 [file nutrients-09-01051-s001.zip › nutrients-219024-supplementary.pdf]

Jaksevicius et al: Supplementary Figures

(a)

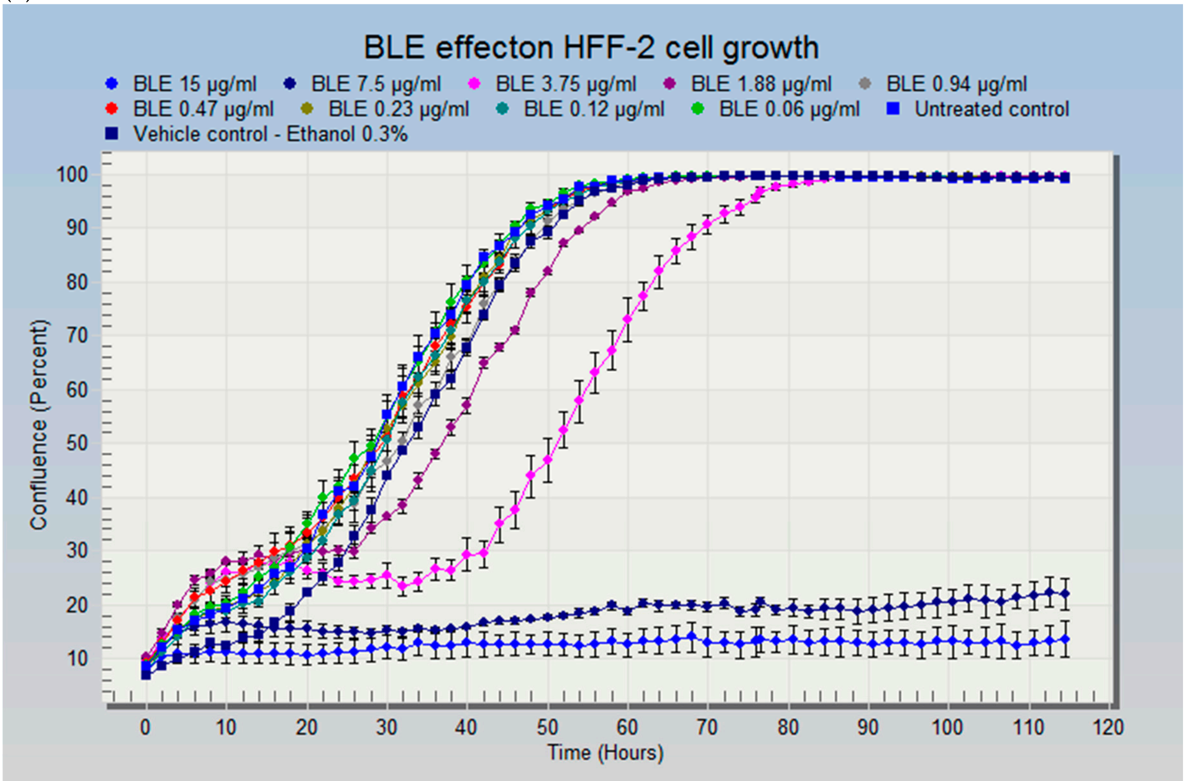

(b)

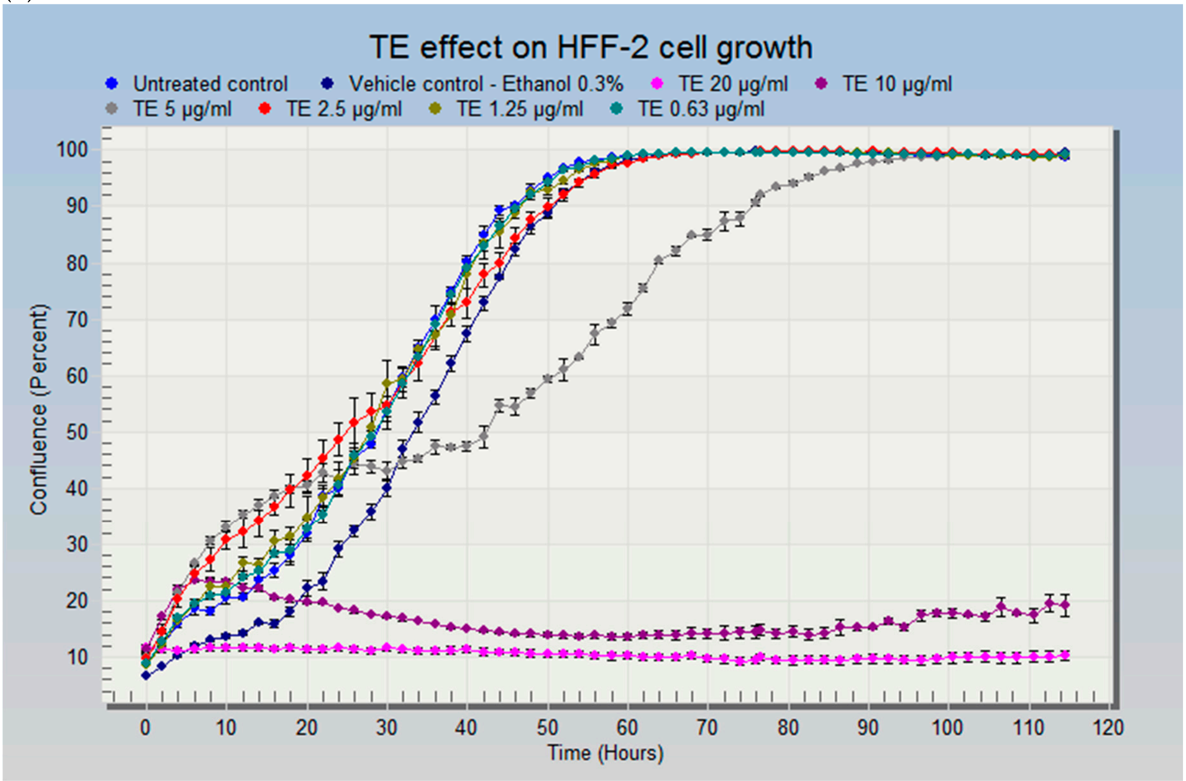

Figure S1. (a) BLE effect on HFF-2 cell growth. **(b)** TE effect on HFF-2 cell growth. Cells were treated with a range of concentrations and the growth was monitored using IncuCyte camera. Data were analysed and presented using IncuCyte software (Essen Bioscience, UK).

(a)

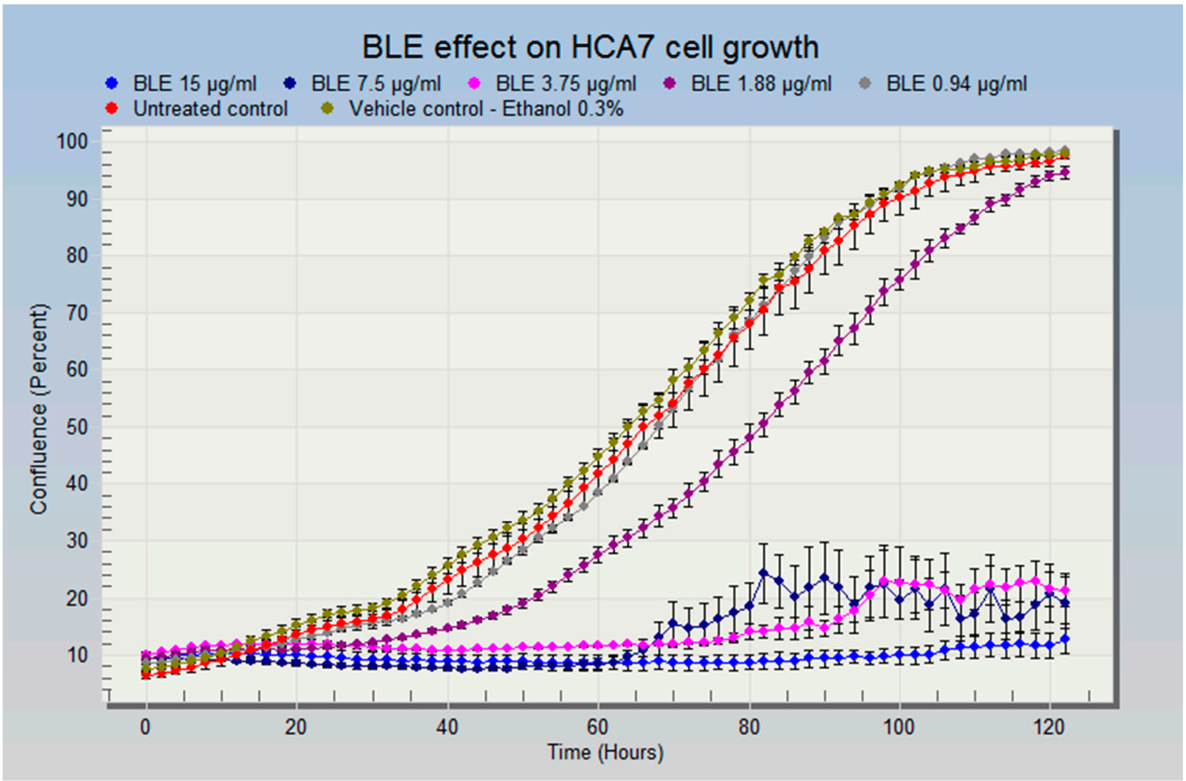

(b)

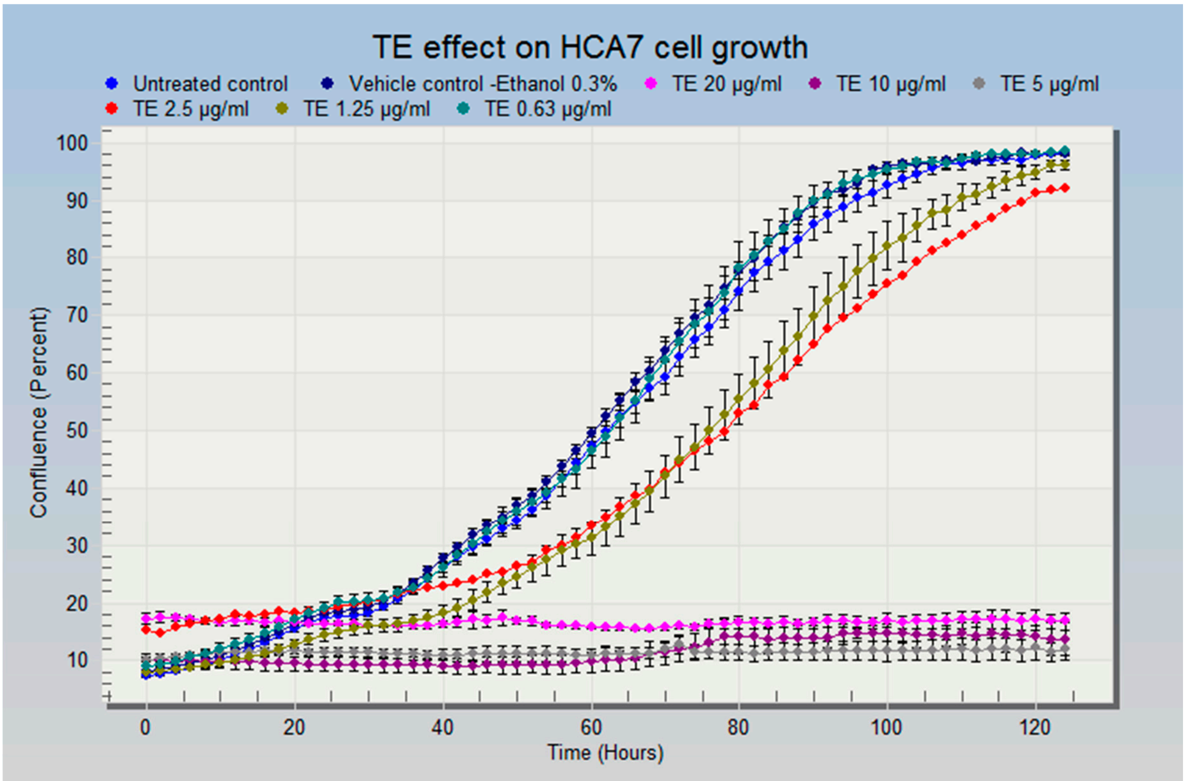

Figure S2. (a) BLE effect on HCA-7 cell growth. **(b)** TE effect on HCA-7 cell growth. Cells were treated with a range of concentrations and the growth was monitored using IncuCyte camera. Data were analysed and presented using IncuCyte software (Essen Bioscience, UK).
